# The end of the line: competitive exclusion and the extinction of historical entities

**DOI:** 10.1098/rsos.221210

**Published:** 2023-02-22

**Authors:** Luke C. Strotz, Bruce S. Lieberman

**Affiliations:** ^1^ State Key Laboratory of Continental Dynamics, Shaanxi Key Laboratory of Early Life & Environments and Department of Geology, Northwest University, Xi'an 710069, People's Republic of China; ^2^ Biodiversity Institute and Department of Ecology & Evolutionary Biology, University of Kansas, Lawrence, KS 66045, USA; ^3^ Department of Palaeontology, University of Vienna, Althanstrasse 14, 1090 Vienna, Austria

**Keywords:** competition, character displacement, competitive displacement, material culture, macroevolution

## Abstract

Identifying competitive exclusion at the macroevolutionary scale has typically relied on demonstrating a reciprocal, contradictory response by two co-occurring, functionally similar clades. Finding definitive examples of such a response in fossil time series has proven challenging, however, as has controlling for the effects of a changing physical environment. We take a novel approach to this issue by quantifying variation in trait values that capture almost the entirety of function for steam locomotives (SL), a known example of competitive exclusion from material culture, with the goal of identifying patterns suitable for assessing clade replacement in the fossil record. Our analyses find evidence of an immediate, directional response to the first appearance of a direct competitor, with subsequent competitors further reducing the realized niche of SLs, until extinction was the inevitable outcome. These results demonstrate when interspecific competition should lead to extinction and suggest that clade replacement may only occur when niche overlap between an incumbent and its competitors is near absolute and where the incumbent is incapable of transitioning to a new adaptive zone. Our findings provide the basis for a new approach to analyse putative examples of competitive exclusion that is largely free of *a priori* assumptions.

## 1. Introduction

The import of interspecific competition in both evolution and ecology has long been recognized [1,2], but the capacity for biotic interactions at the organism and population level to impact directly upon species richness remains an open question [3–9]. With multiple putative examples of clade replacement identified in the fossil record [5,10], considerable attention in palaeontology has been paid to the possible role competition plays in driving extinction [11], although a diminished role for competitive interactions in macroevolution has also been proposed [12]. Uncovering the relative significance of antagonistic interactions in extinction is a topic that is relevant across the biological sciences. For instance, in community ecology, the possibility of unconstrained competition-driven extinction would rationalize limits on global biodiversity [6,13], suggest a relationship between species survival and species longevity [8,14], and explain the relative frequency of competitive exclusion [15]. As competition operates at the level of populations within communities, the capacity for interspecific competition to engender extinction also supports the possibility of a continuum between phenomena at the level of populations through to the level of the species [16].

The challenge of recognizing or refuting interspecific competition as a primary driver of extinction arises partly from the difficulty of identifying possible macroevolutionary examples that can be substantiated or refuted. While a reciprocal, contradictory response by two functionally similar and overlapping clades has typically been invoked as evidence of antagonistic clade displacement, confirming that a correlative or causal relationship exists between time series remains problematic [9]. Entirely divorcing such an interaction from abiotic pressures associated with ecological niche is also an elusive prospect at the macroevolutionary scale, due partly to an absence of abiotic constancy at any time in the geological record [17]. With the fossil record representing the primary evidence of extinction dynamics, temporal and preservation limitations that often hamper recognition of competition and any associated response may also impede identification of competitive exclusion [9,18].

We propose several key prerequisites (table 1) needed for any macroevolutionary system to be suitable for identifying interspecific competition as the primary cause of any specific extinction: (*i*) functional trait data relevant to survival of individuals within the extinct clade can be quantified; these data are important, as existing theory predicts displacement of traits that impact resource use due to competition between one or more overlapping species [19,20]; (*ii*) direct competitors have been identified that overlap spatially, temporally and in their resource requirements [12]; (*iii*) a complete (birth to death), well-constrained time series is available for the extinct clade, where the vast majority of individuals that make up the extinct clade are known; at a minimum, the date of first appearance for relevant competitors must also be known; and (*iv*) non-competitive factors (which for a biological organism would constitute changes in the abiotic realm) do not apply or are irrelevant. Arguably, identifying fossil clades that adhere to these four criteria ranges from highly refractory to impossible, with criterion (*iv*) particularly problematic.

**Table 1.** Proposed criteria to identify interspecific competition as the primary cause of a specific extinction. Where an extinction is considered in isolation and all four criteria cannot be met, interspecific competition cannot be confirmed as the cause of extinction.

*i*.	functional trait data relevant to survival of individuals within the extinct clade can be quantified
*ii*.	direct competitors have been identified that overlap spatially, temporally and in their resource requirements
*iii*.	a complete (birth to death), well-constrained time series is available for the extinct clade, where the vast majority of individuals that make up the extinct clade are known
*iv*.	non-competitive factors do not apply or are irrelevant.

While there have been previous attempts to generate theoretical predictions of the expected patterns arising from interspecific competition [20,21], more empirical data is needed to assess the relevance of these to presumed macroevolutionary examples of competition. Microevolutionary examples also provide a potential analogue, demonstrating that biotic interactions can result in directional shifts in functional trait space [20,22]. Directional selection driven by competition does not necessarily indicate inevitable extinction, however, and for such examples to retain relevance in a macroevolutionary context also requires a linear hierarchy between micro- and macro-evolutionary processes, a debate that is far from resolved [8,10,11].

Competition and extinction are phenomena that extend beyond the biological realm and previous studies have demonstrated how both can apply to well-recognized commercial, cultural and technological entities [23–27]. This presents the possibility of examining a known competition-facilitated extinction event that occurs at a scale equivalent to macroevolutionary processes as a means of identifying the expected patterns associated with competition-mediated extinction in the fossil record. One candidate example is the steam locomotive (SL), which became effectively extinct as competition made continued production no longer economically viable [28,29]. Representing one of the most transformative technologies in human history and the first significant advance in land transport since the domestication of the horse and the development of the wheel thousands of years prior [30], SLs were the dominant technology for transport of goods and passengers through the nineteenth and early twentieth centuries. Given their functional traits can be precisely quantified and their extinction is definitively attributed to the development of a known pool of direct competitors with superior properties [30–32], patterns of change in functional values for SLs resulting from competitive forcing offer the opportunity to shed light on how competition may lead to extinction for a variety of historical entities, including macroevolutionary taxa.

We assess the impact of direct competition on the SL throughout its lifespan with the aim of identifying diagnostic patterns relevant to presumed examples of competitive exclusion and associated extinction in the fossil record. After first verifying how SLs satisfy our criteria (*i–iv*) for identifying interspecific competition, we consider functional trait data for SLs using a quantitative framework, evaluating if functional trait values exhibited directional shifts as competitors first appeared, or whether any shifts are better explained by other factors considered relevant to SL production but not related to competition. We examine these results in light of the principles of competitive exclusion and conclude with a discussion of the implications of our results for macroevolutionary studies.

## 2. Evaluating criteria (*i–iv*) for steam locomotives

The primary function of a locomotive is to move a train of railroad cars, whether that train be composed of goods wagons (freight) or passenger coaches. To do so, a locomotive must generate sufficient force to overcome the drag imposed by the train it is pulling or pushing. The force a locomotive is capable of generating is encapsulated by a parameter known as tractive effort (TE) [33,34]. Because TE reflects the maximum train weight a locomotive can put into motion and the maximum speed it can reach while doing so, a direct link exists between TE values and locomotive productivity [35] and the life-cycle cost of an individual locomotive is correlated with its maximum TE value [33]. The maximum tractive effort value for any individual locomotive thus captures *virtually the entire function of that individual*, demonstrating criterion *(i)* is applicable to SLs.

Production and maintenance of any land transport technology will only continue while the benefit-to-cost ratio for that technology to move passengers and/or freight is equal to or greater than existing alternatives [32]. Any competitor of SLs would thus take the form of alternative technologies that could transport the same or greater volume of passengers and/or freight with greater efficiency or at a reduced cost. Three technologies fulfil this requirement. Two of these technologies, electric and diesel locomotives, exhibit near total niche overlap with SLs and represent unambiguous competitors, validating criterion (*ii*). All locomotive types compete for the same primary resource (passengers and freight), their capability to do so is captured by the same functional trait (TE) and spatial overlap is total, all utilizing railroad track, with the caveat of overhead wires or an electrified third rail as an additional requirement for electric locomotives. The third competitor for SLs is motor vehicles (e.g. automobiles and trucks) [36]. Despite significant differences in carrying capacity between motor vehicles and SLs, the primary function of motor vehicles is still movement of passengers and/or freight, and tractive effort is a relevant parameter for determining the capacity of a motor vehicle to fulfil this role [37]. A decline in rail passenger traffic in both the United States and United Kingdom in the first half of the twentieth century coincides with an increase in motor vehicle production and motor vehicle registration in both those countries (electronic supplementary material, figure S1).

When attempting to determine if interspecific competition is a primary driver of an extinction event, it is necessary to establish overlap between the competing clades and demonstrate a relevant transformation in the clade (such as character displacement) that has putatively been driven to extinction by competitive forces during the period of overlap, i.e. criterion (*iii*). Issues such as provincialism, preservation potential and taxonomic inconsistencies can make overlap difficult to establish for fossil taxa [38]. By contrast, yearly production and usage time-series data are available for SLs and their competitors in publicly available databases (electronic supplementary material, figure S2), fulfilling criterion (*iii*).

Criterion (*iv*)'s veracity in the case of SLs is well supported by historical data (e.g. [30–32]). Non-competitive factors with the potential to drive SLs to extinction include (1) the loss of the technical expertise necessary for their construction, (2) a diminished need to move passengers or freight overland, and (3) increasing scarcity of the resources needed for their construction and/or operation. We can easily dismiss the first possibility, as the expertise needed to construct new SLs exists presently [34]. For the second possibility, during the temporal range used in our study, the total tonnage of freight moved by rail actually increases (electronic supplementary material, figure S3). Rail passenger numbers do decline in both the United States and the United Kingdom in the first half of the twentieth century, but this coincides with a marked increase in motor vehicle usage (electronic supplementary material, figure S1). Rail passenger numbers in many mainland European countries, where electric locomotives are common [39], increase over the entire study period [40]. Demand for land transportation, either by rail or overall, has thus not declined. In regard to the third possibility, both SLs and their competitors use the same resources for construction and maintenance, and those resources remain in ready supply [41]. A primary difference between SLs and other land transportation alternatives is that steam locomotives require solid fuel to generate tractive effort [42]. Coal has never been in short supply during the lifespan of the SL, with the current global reserve-to-production ratio for coal equal to almost 140 years [43], and the price of coal, in real terms, remaining unchanged for the entire interval of competition (electronic supplementary material, figure S4).

While we provide evidence that SLs adhere to our four criteria, we do not propose that a technological entity can be considered truly homologous with a macroevolutionary unit [23–25]. For instance, SLs cannot be considered monophyletic and they are incapable of speciation. We do consider them strongly analogous, however, as they do experience both ‘birth’ and ‘death’, and are subject to processes that fall within the purview of macroecology. In this sense, technological entities can be considered parallel to higher biological units, such as genera and above [44]. Although the functional lifespan of the SL is several orders of magnitude shorter than typical macroevolutionary units, the rapid generation time of new locomotive forms (measured on a scale of months/years) means SLs can be considered comparable to a ‘temporally condensed’ macroevolutionary unit.

An additional proviso is that, in the biological sense, SLs are not strictly extinct. It remains possible to construct new SLs [34] and operational SLs currently exist [45]. They are, however, effectively extinct, no longer either a subsidiary component of the land transportation network nor an economically sound means of transportation [32]. Following World War 2 (WW2), production of new steam locomotives ceased almost entirely (electronic supplementary material, figure S2). Mainline operations using SLs continued in some countries into the second half of the twentieth century but did so using older locomotives [39]. China recently terminated all mainline operations utilizing SLs, the last country to do so [46]. Extant SLs have now been reduced to the role of a ‘curiosity’, used for entertainment or as functioning museum pieces. With production of SLs effectively zero, the SL is much more than a ‘dead clade walking’ [47]. It is as ‘extinct’ as any technology can be, given true extinction of any technological entity would generally require the concomitant extinction of humans.

## 3. Material and methods

The TE values used to test for character displacement come from *Locobase* [48], a database that provides specifications and production data for almost every SL ever built (approx. 16 000 locomotive types). We define a type of locomotive as an individual design, where multiple individual locomotives may have been built using that same design. Values for locomotives that were never actually put into production or that fell outside the temporal range of our study were pruned from the total dataset. The first year of our time series is 1829, coincident with the construction of *Stephenson's Rocket* [30]. While *Stephenson's Rocket* may not represent the first SL built, it is the first to possess all the constituent parts standard to subsequent SL designs [30]. The vast majority of SLs ever produced were constructed in Europe and North America [48], and the last locomotive for mainline usage produced in these two regions was completed in 1960 [49]. *Locobase* lists 1964 as the last year where multiple new types of SL were constructed, and it is reasonable to use this year as the last year of our time series, given that all major producers had ceased production by this date.

For each year, from 1829–1964, we calculate the mean, maximum, minimum and mode for TE to construct a time series for each of these parameters. Maximum and minimum values represent the single maximum or minimum TE value, respectively, across all types of SL for a given year. As TE represents a continuous variable, we employ kernel density estimation to estimate a modal value, using the peak value as a proxy for the mode. All values for time series were calculated using *base* R [50].

**Figure 1.**
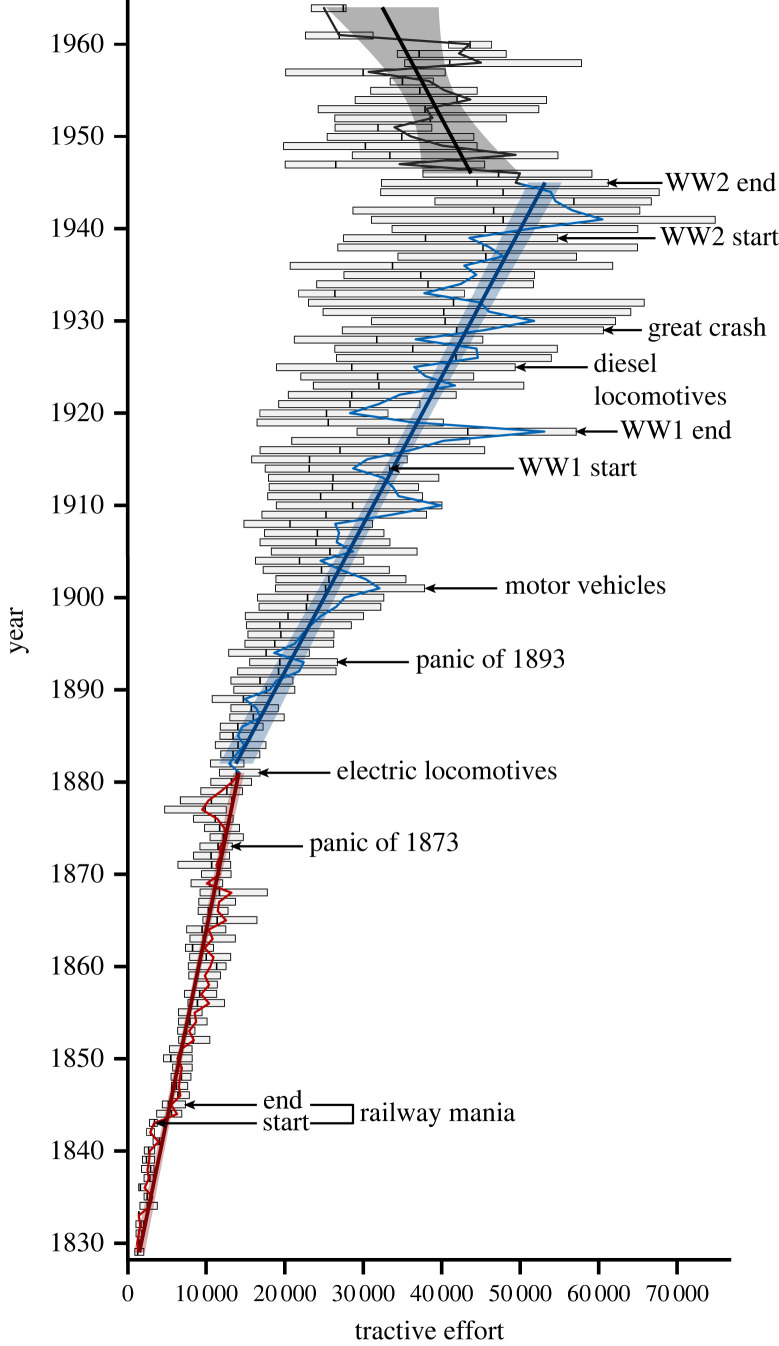
Mean tractive effort for steam locomotives 1829–1964. Three phases are identified for mean TE based upon multivariate adaptive regression splines (MARS) analysis. Red = 1829–1881; blue = 1882–1945; black = 1946–1964. For each year in the time series, both mean value (solid fluctuating line) and the interquartile range of TE values for that year are provided, calculated using *base* R [50]. Solid straight lines represent linear regression lines for each phase, with shaded areas signifying 95% confidence intervals. The timing of the first appearance of competitors and other contingent events are marked as indicated.

To determine the first appearance dates for known SL competitors (criterion *ii*), we employ the simplifying assumption that competition was initiated once the competitor was produced/employed in a commercial context or readily acquired in the marketplace, and thus actually competing with steam locomotives for the same resources (transportable passengers and freight). In the case of electric locomotives, a term used herein to capture all forms of electrified light and heavy rail, we use the completion of the first electric tramway constructed in a major metropolitan centre, the Gross-Lichterfelde Tramway, which began service in 1881 [51]. For motor vehicles, we use the beginning of the Oil Age and the introduction of the Curved Dash Oldsmobile automobile, considered the first mass-produced and affordable automobile, both of which occurred in 1901 [52]. For diesel locomotives, we use delivery of the GE-IR-ALCO boxcab in 1925, the first diesel locomotive constructed for commercial purposes [32]. We also include in our assessment key historical or contingent (*sensu* [53]) events that conceivably altered the competitive landscape (figure 1). These include global-scale economic depression events (the ‘panics’ of the nineteenth and early twentieth century) and the two World Wars (WW1 and WW2).

Initial assessment of TE data was undertaken by generating hinge functions using multivariate adaptive regression splines (MARS), a non-parametric regression method developed to model high-dimensional nonlinear data while avoiding overfitting [54]. With no *a priori* assumptions about the form of nonlinearity, MARS can serve as a neutral model to identify potential changes in trend direction and slope. Because a number of cut-points in the resulting hinge function may contribute little to model accuracy, model fit was assessed using *k*-fold cross-validation (*k* = 10), with the asymptote of R^2^ used to determine the number of knots to be retained. MARS models were implemented using the *earth* package for R [55].

Random walk models previously applied in macroevolutionary studies [56–58] provide a means to identify directional change. We evaluate whether the time series between each knot is best characterized by one of four models: an unbiased random walk (defined as a generalized random walk with a zero mean step size); directional change (defined as a generalized random walk with a non-zero mean step size); stasis (defined as uncorrelated, normally distributed variation around a steady mean); and strict stasis (instances where the variance around the long-term mean is zero, such that there is no change between samples) [57]. Models were fit by maximum likelihood using the *paleoTS* package for R, using the standard protocols outlined by Hunt [56,57]. Because these models require mean values, variances, sample sizes and relative ages [56,58], this analysis could only be performed on mean TE data. Support for each model was assessed using the bias-corrected Akaike information criterion (AICc). The lowest AICc value was considered to be the best-supported model, and the probability that any one model is the best supported was determined using Akaike weights [59].

To assess how results for SLs may inform analyses of competitive exclusion for fossil taxa, maximum, mean and minimum body size for the Borophaginae, a subfamily of the Canidae purported to have been competitively displaced in North America by multiple carnivore clades, were calculated using data from [60].

## 4. Results and discussion

**Figure 2.**
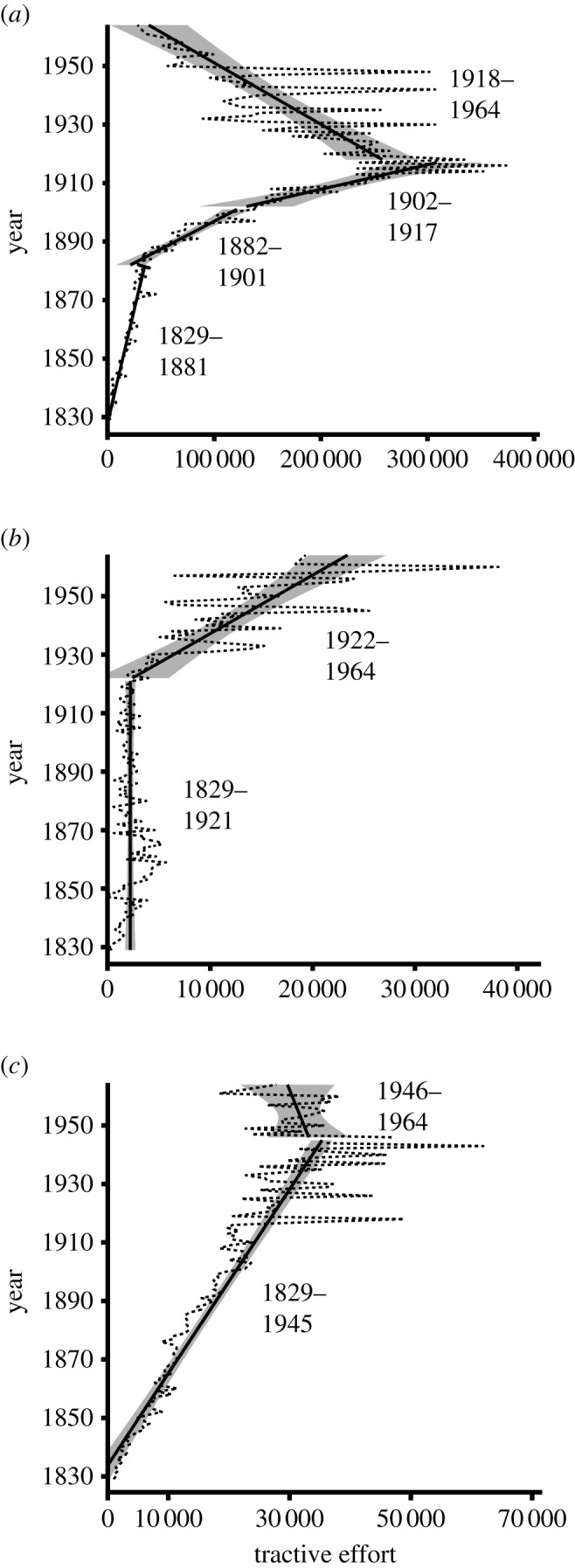
Maximum, minimum and modal values for TE 1829–1964. (*a*) Maximum TE; (*b*) minimum TE; (*c*) modal TE. Temporal phases designated on each figure identified using MARS analysis. Solid straight lines represent linear regression lines for each phase, with shaded areas signifying 95% confidence intervals.

The appearance of direct competitors results in measurable displacement of SL functional trait values (figures 1 and 2). The most conspicuous is the shift in trend slope and mode of trait change for mean TE coincident with the first appearance of electric locomotives. MARS identifies a cut-point for mean TE at 1881, with a steep increase in trend slope following the cut-point (figure 1). Prior to 1881, mean TE values are best characterized by an unbiased random walk, and values after the first appearance of a direct competitor are best represented by directional change (table 2). Simultaneously, there is an increase in trend slope for maximum TE (figure 2*a*). These shifts can be attributed to increasing electrification of suburban commuter routes, where lower TE values were sufficient, and increasing demand for high-speed intercity passenger and freight services, tasks both requiring higher TE values [29].

**Table 2.** Maximum-likelihood parameter estimates for mean TE values. AICc values and Akaike weights for four models: GRW (general random walk), URW (unbiased random walk), Stasis and S. Stasis (strict stasis). Definition for each model provided in Materials and methods. Values rounded to three decimal places.

	AICc				Akaike weight			
	GRW	URW	Stasis	S. Stasis	GRW	URW	Stasis	S. Stasis
1829–1881	974.1	899.25	1033.228	5735.203	0.000	1.000	0.000	0.000
1882–1945	1257.02	1535.653	1389.747	5310.084	1.000	0.000	0.000	0.000

The emergence of subsequent competitors initially had a lesser impact, with only an increase in the rate of positive change for maximum TE coincident with the first appearance of the motor car (figure 2*a*). The cut-point for minimum TE in 1921 (figure 2*b*), however, is probably due to competition from motor vehicles. Rail passenger numbers in North America and Europe sharply declined in 1921, corresponding with a rise in motor vehicle usage (electronic supplementary material, figure S1). This sudden preference for motor vehicles is due to the development and expansion of highway networks in the 1920s [61], allowing motor vehicles to be employed for all-weather travel between urban centres. The decline in mean and modal TE from 1945 onwards (figures 1 and 2*c*) also probably represents a lagged response, specifically to the dieselization of rail networks. Diesel possessed multiple advantages over steam [32], but war production controls and petroleum shortages associated with WW2 hindered a mass roll-out prior to 1945 [28]. With these restrictions released, diesel quickly became the primary locomotive technology [32].

The peak in maximum TE coincides with nationalization of US railroads during WW1 [29]. Nationalization stifled production for the largest and most innovative SL producers at the time, restricting production to smaller designs with lower TE values than were possible at that point [62]. It was clear, however, even prior to nationalization, that the limits of TE for SLs were being reached. Couplers could not withstand the strain generated by the most powerful locomotives, the largest locomotives had clearance issues when travelling through tunnels and some of the most extreme examples were only fit for use when subsequently broken down into two separate units [63]. SLs had thus reached their ‘right wall’ (*sensu* [53,64]) and size limit (*sensu* [65]), with TE unable to increase further due to physical and practical constraints [32].

Unlike competitive factors, non-competitive contingent events had limited impact on SL TE values. Economic depressions caused only brief declines in mean TE and both World Wars coincide with a sharp, but short-lived, increase in mean TE (figure 1). These events reduced or increased the demand placed on transport networks or impacted on the viability of competitors, but their effect was ephemeral. As such, they can be considered equivalent to a form of ecological disturbance, where community structure is disrupted but rapidly returns to its pre-event form once the disturbance has passed [66]. For example, SLs were essentially equivalent to a disaster taxon [67] during WW2, as increased demand and petroleum shortages forced an upsurge in SL production (electronic supplementary material, figure S2). While this temporary increase in demand prolonged the lifespan of the SL [28], production returned to pre-war values once hostilities ceased (electronic supplementary material, figure S2) and the SL was soon thereafter functionally extinct.

**Figure 3.**
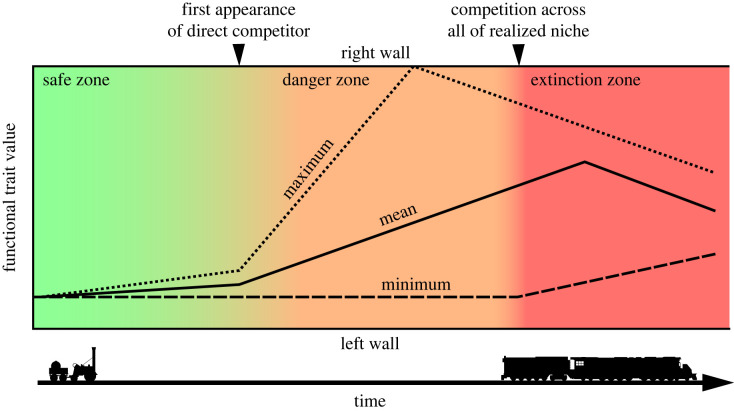
Graphical depiction of expected pattern when interspecific competition is the primary driver of extinction, based on results from steam locomotives. Early in its history, the incumbent is in the ‘*safe zone*’, with neutral drift in functional trait space away from a bounding ‘left wall’ resulting in an inexorable slow rise in both maximum and mean values. With the first appearance of a direct competitor (first arrowhead at top), character displacement is initiated and there is a directional shift in the mean and rapid increase in the maximum. The incumbent is now in the ‘*danger zone*’, as competitors siphon off part of the available resource pool and reduce the extent of the incumbent's realized niche. Unless competition remains restricted or the incumbent can reach a new adaptive zone, the likelihood of extinction continues to increase. Extinction is neither imminent nor inevitable at this stage, because at least some populations at the fringe of the optimal fitness landscape remain shielded from competition or are at a competitive advantage, as indicated by the still stable minimum value. If competition drives the incumbent to seek a new fitness optimum and it is unable to reach a new adaptive zone, one or more populations will collide with the ‘right wall’, where functional trait values cannot rise any further due to physical or practical constraints. At this point, the capacity for the incumbent to expand its realized niche is compromised and the incumbent may even ‘bounce’ off the right wall, as the most extreme populations are eliminated due to low fitness. Extinction is still not guaranteed at this stage, as the stable minimum continues to indicate populations of the incumbent exist that are unaffected by competition. It is only when the minimum functional trait value detaches from the ‘left wall’ that all populations of the incumbent are subject to competitive exclusion, and the incumbent enters the ‘*extinction zone*’. The available realized niche space for the incumbent rapidly declines as pressure is exerted across all parts of its fitness landscape. While persistent disruption may prolong the lifespan of the incumbent, by providing the incumbent with a temporary competitive advantage, any such reprieve will only last as long as the disruptive event. Extinction of the incumbent at this stage is inevitable.

The results of our analyses provide the basis of a model suitable for identifying when interspecific competition is the primary driver of extinction (figure 3). In the absence of substantial environmental change and competition, an incumbent clade would be expected to diversify in a drifting or neutral fashion [68,69]. Mean and median functional trait values will necessarily increase in such a scenario, as trait values are limited by a bounding ‘left wall’ (*sensu* [53,64]), with the pattern of change corresponding to an unbiased random walk. Our results (figure 1) indicate that the appearance of direct competition can compel directional adaptation on such a system, possibly due to a form of niche differentiation [70] through resource partitioning [71]. Because an unlimited increase in functional efficacy is impossible due to physical limits, failure to transition to new adaptive niche results in reaching the functional ‘right wall’, an associated reduction in variation [53], and even the possibility of ‘bouncing’ off the right wall (figure 3). If populations at the fringe of the optimal fitness landscape are then also competitively excluded, represented by the minimum functional trait value detaching from the ‘left wall’, the realized niche occupation for the incumbent clade will rapidly decline. With a positive relationship between realized niche breadth and survivorship [72], extinction becomes the inevitable outcome (figure 3).

We do not mean to imply that the above scenario represents the only means by which competitive exclusion can drive a clade to extinction, or that competition for the same resource pool must result in extinction. Support for character displacement is generally confined to animal groups, specifically carnivores [73] and the absence of competitive exclusion in terrestrial plant communities forms the basis for theoretical treatments that devalue the role of competition in ecology [74,75]. The strength of the signal we observe could therefore be viewed as reinforcing Gause's [76] original definition of competitive exclusion: that species with *identical* niches cannot coexist indefinitely. The extinction of the SL may only have come to pass because the niche overlap between steam and competing technologies far exceeds the maximum allowable overlap for two clades to coexist [74] and SLs were incapable of transitioning to a new adaptive zone [8]. Figure 3 can thus be considered a form of neutral model, as a scenario where competitive forces are at their utmost and where abiotic factors are absent will rarely exist in nature. As with all neutral models, deviations away from this extreme scenario allow the relative import of other drivers to be quantified. For example, a failure to find compelling evidence for competitive exclusion in the fossil record [9] may thus reflect the possibility that resource competition is more diffuse in biological systems than in material culture, diluting the impact of interspecific competition and limiting the possibility of extinction as a result of biotic interactions.

The proposed model we present herein both establishes that the impact of competition on functional traits can be essentially immediate and provides a previously unlooked-for pattern to investigate clade replacement in the fossil record by focusing on functional traits that can be measured for fossil organisms, such as body size and metabolic rate [77]. The immediate response that we identify in the incumbent clade to the onset of competition suggests that there need not be an expectation of significant lag in the response for fossil examples, barring issues with establishing temporal coexistence [38], if the species are competing for the same resources. Importantly, our proposed approach is largely agnostic, not requiring that a competitive relationship be verified *a priori*, but only that a putative one exist. While directional change in the mean trait value need not be indicative of interspecific competition when identified in isolation, when considered in light of the full spectrum of variation [53], such change can provide robust evidence for competitive exclusion (figure 3). It is unlikely that, for many groups, the near-entirety of function for a biological clade can be captured by a single trait, as is possible for SLs. Previous work has demonstrated, however, that even for a very large clade, a very small number of traits can be representative of the variation in growth, survival and reproduction for that clade [78]. Multiple functional traits can be considered in isolation using our approach, or multivariate data can be reduced to a single value using analytical techniques that condense the number of dimensions in the data (principal component analysis, factor analysis, etc.) while still preserving as much of the variation in the data as possible [66].

Comparison of our results with a fossil example of putative competitive exclusion, specifically competition between subfamilies in the Canidae in North America, affirms that the patterns we identify for SLs can be observed in natural systems where competition between clades is considered significant [60]. The onset of character displacement for the Borophaginae coincides with the first appearance of a direct competitor and increased displacement of functional trait values corresponds to both the emergence of new competitors and an increase in niche overlap for the competing clades (electronic supplementary material, figure S5). It is important to recognize though that in either biological or cultural systems, there may also be several different factors beyond competition that explain any observed variation [79]. It should also be noted that the relevant data needed to conduct a full assessment of both the frequency of our proposed model in the fossil record and the relative import of clade replacement in macroevolutionary dynamics is currently lacking, as the necessary functional trait data associated with putative examples of competition-driven extinction has not yet been compiled. The fossil record does, however, contain a plethora of suitable options from which such data could be obtained.

Our results thus cannot, at this time, resolve what role, if any, competition plays in driving extinction at the macroevolutionary scale. They do, however, provide a path forward that may serve to resolve the issue by addressing the existing impasse in identifying causal relationships in fossil time series [9]. There is no doubt that the SL was derailed by its competitors; however, it still remains to be established if biological clades are frequently similarly sidetracked.

## Data Availability

The data analysed in this study come from *Locobase*, available at http://steamlocomotive.com. Both the tractive effort data and the R script used to generate the results presented in this paper are available on Dryad Digital Repository: https://doi.org/10.5061/dryad.w9ghx3ft2 [80]. All supplementary data was sourced from publicly available published works (see relevant citations). Supplementary figures are available in the electronic supplementary material [81].
